# Problem-based learning sessions and undergraduate research: a medical student’s perspective and experience

**DOI:** 10.1007/s40037-013-0069-9

**Published:** 2013-07-11

**Authors:** Abdulhadi A. AlAmodi

**Affiliations:** College of Medicine, Alfaisal University, Riyadh, 11533 Kingdom of Saudi Arabia

**Keywords:** Problem-based learning, Undergraduate research, Curriculum, Education

## Abstract

Undergraduate research (UR) and problem-based learning (PBL) sessions are similar with respect to the type of skills gained through each. However, appropriate modification of PBL sessions would contribute to enhanced UR experience. Based on personal experience in UR and a PBL curriculum, in this short discourse I shall explain how studying under a PBL curriculum enabled me to gain more out of my research experience.

## Introduction

The definition of undergraduate research (UR) is perceived differently by universities worldwide [1]. In the USA, it is viewed as an investigation conducted by an undergraduate student which is culminated by knowledge dissemination [2]. On the other hand, other institutes see UR as a tool through which the learning and teaching environment may mimic research-based skills [1]. However, it is widely accepted that within this diverse spectrum, undergraduate students should develop competencies and skills which will help them deal effectively with intricate problems in the discipline [3]. In this context, there is a debate between modernists and traditionalists on determining the best pedagogy to teach medical students—especially with the current complexity and expansion in knowledge, technology and specialization [4]. This dilemma is intensified especially with regard to adopting the problem-based learning (PBL) curriculum in medical schools. However, many medical schools continue to shift toward implementing the PBL curriculum [5]. The rationale includes that PBL supports the application of fundamental learning skills such as communication and critical thinking skills, as well as integrating a wide range of basic concepts in biomedical sciences [6].

Alfaisal University College of Medicine is located in Riyadh—the capital city of the Kingdom of Saudi Arabia. The institute is a 5-year-old, private, non-profit organization that supports and encourages its undergraduate students to be engaged in research early on in medical school. For that reason, it has set up an Undergraduate Research Committee (URC) which facilitates students’ contributions in high-quality collaborative research in local and international research centres. Also, it provides students with scholarships to run their projects, present in professional meetings and participate in international summer research opportunities. Moreover, the institute adopts the PBL curriculum as the core modality in teaching medical students. In this short discourse, I describe my personal experience in dealing with two pedagogies—PBL and UR—and how PBL can be utilized effectively to result in better UR experience.

## Personal experience in physiology and biophysics research

I was selected by the Summer Programme for International Research Internship and Training (SPIRIT) at Alfaisal University College of Medicine to conduct basic biomedical research in the Department of Physiology and Biophysics, Center for Excellence in Cardiovascular-Renal Research, University of Mississippi Medical Center (UMMC) (USA) for 5 months over two consecutive summer vacations. My laboratory studies the role of haemeoxygenase-1 and its metabolites, carbon monoxide and bilirubin, in regulating fat metabolism and blood pressure. This experience has promoted my interest in basic biomedical research and empowered me to realize the importance of utilizing the PBL sessions effectively.

## Discussion

In my opinion, an effective educational environment is one which facilitates students’ transition from individuals who consume knowledge to those who possess the necessary skills to contribute to, and produce, knowledge. Recurrent exposure to research in laboratories is a potent means to achieve this prime goal. Generally, conducting research has shown to increase students’ chances of employment, enhance postgraduate research and create non-isolated physicians [7, 8]. On a personal scale, as a consequence of spending an average of 35 h during a period of 20 weeks over two consecutive summers engaging in basic biomedical research in the laboratory at the UMMC, I was fortunate enough to be equipped with skills which would require much more time to develop within traditional classrooms. These skills include deciphering and optimizing scientific protocols, running experiments using in vitro and in vivo techniques, generating data, evaluating its reliability and conducting data analysis, dealing with troubleshooting in experiments, integrating and correlating data generated from different experiments, questioning and proposing further ideas to be studied, developing communications skills by presenting updates to my mentor and deciding on what to do next. This dynamic process enabled me to gain hands-on basic research experience, as well as advocating better personal appreciation of how knowledge is generated via research. Moreover, the experience also advanced my ability to criticize and critically evaluate presented knowledge.

Of note, I was able to appreciate that the mentor plays an important role in maximizing students’ benefits and gains. A supportive mentor, like Professor David E. Stec, encourages students to develop independency and confidence over time, giving them ownership of the project when deserving of it, providing useful feedback and treating dedicated deserving students as quasi researchers.

Similarly, I would expand and label PBL sessions as the ‘small laboratory’ because they assisted me to practice similar skills at a lesser scale, which I was able to easily build upon during my research experience. These PBL sessions are structured based on clinically based problems. PBL reinforces the application of hypothesis-driven reasoning by allowing students to deal with a problem (‘Chief Complaint’), analyzing the problem and acquiring further data about it (‘History of Present Illness’), suggesting appropriate clinical laboratory tests to be ordered and providing the rationale (‘Investigations’), deciding on how to move from one laboratory investigation to another based on the amount of knowledge acquired at each stage (‘Plan’), making use of literature to figure out answers for outlined objectives and knowledge deficiency, and lastly, presenting and communicating findings to the student team and PBL facilitator. Furthermore, the most important task within the PBL setting that helps to improve high cognitive skills is perceiving information from different laboratory results such as radiology, histopathology and biochemistry, and associating it with the clinical picture presented in the PBL case. Last but not the least, setting up the management plan based on all accumulated and integrated knowledge acquired from the patient’s history, physical examination and laboratory data, greatly helps students to broaden their understanding of the subject at hand, honing and enhancing any skills acquired during the course of the session/s.

Despite conducting my research project away from home, which can be challenging [9], the basic skills I had acquired from my PBL sessions at the medical school in the KSA were so similar to basic laboratory research practice dynamics that it was not exceedingly difficult or impossible to cope and adapt to the new learning environment; I was able to function in the laboratory with confidence and further enhance the subset of skills I had learned from the PBL sessions at medical school.

PBL can be a very effective learning environment for cognitive operation. From my perspective, there are basic requirements and criteria which need to be met to facilitate such environment—most of which I experienced first-hand at my institute. Firstly, PBL scenarios should not be given completely on the first day; rather the scenario (clinical case) should be broken into parts, and before moving into each part, there should be constant emphasis on students’ opinions about ‘what is required next’. Also, PBL scenarios should be structured to allow integration of various domains such as knowledge, attitudes and skills. Secondly, PBL facilitators should be specialized, research-oriented faculty members who excel at the role of encouraging mentors, developing insight about students’ performance and progress and using it to facilitate each student’s skills and capabilities as required. This can be achieved by maintaining a fair faculty/students ratio, giving faculty the chance to provide feedback and recommendations to improve to students. Finally, sometimes not all students are interested in actively engaging in discussions because their adopted learning approach differs from the one suitable for the PBL environment; the potential method to resolve this crisis would be modifying students’ attitude and perception toward learning by helping and guiding them to adopt the deep approach to learning which reinforces the integration of various ideas and concepts and stimulates the development of critical thinking skills.

## Conclusion

PBL students’ performance and perception toward basic biomedical research is expected to be more advanced than students studying under non-PBL-based curricula. These two pedagogies—PBL and basic research—are very unique in developing students’ cognitive operations. Efforts are encouraged to promote the transferability of basic skills acquired in PBL to application in conducting research projects.
